# Effectiveness of the Life Enhancement and Advancement Program for Weight Management in Overweight and Obese Females

**DOI:** 10.3390/bs14080724

**Published:** 2024-08-20

**Authors:** Raheleh Maddah Shourche, Mohsen Nematy, W. Miles Cox, Javad S. Fadardi

**Affiliations:** 1 Faculty of Education and Psychology, Ferdowsi University of Mashhad, Mashhad 9177948991, Iran; ra_ma889@mail.um.ac.ir; 2 Department of Nutrition, Mashhad Medical Sciences University, Mashhad 9177899191, Iran; nematym@mums.ac.ir; 3 School of Psychology and Sport Science, Bangor University, Bangor LL57 2DG, UK; m.cox@bangor.ac.uk; 4 School of Community and Global Health, Claremont Graduate University, Claremont, CA 91711, USA

**Keywords:** exercise, dieting, adaptive motivational structure, personal concerns inventory

## Abstract

Background: Obesity has been shown to have many deleterious physical and psychological consequences. Objective: This study examined the effectiveness of adding the Life Enhancement and Advancement Program (LEAP) to a weight management program. Design: To evaluate the different components of a weight loss program, this study included four groups: (1) dieting, exercise, and LEAP; (2) dieting, exercise, and sham training; (3) dieting and exercise; and (4) exercise only. An assessment was administered at baseline, post-intervention, and a three-month follow-up. Participants: Forty female participants with a body mass index (BMI) ≥ 25 were recruited from a local sports center. They provided informed consent and were randomly assigned to one of the four groups. Measures: Participants’ heights were recorded at baseline, and their weight, waist circumference (WC), and BMI were measured at each of the three assessments. Participants completed the Personal Concerns Inventory (PCI) to assess their adaptive motivation at all three assessments. Results: A MANCOVA indicated that participants who received LEAP along with dieting and exercise had a greater reduction in BMI and WC and improved more in adaptive motivation than the other groups. Conclusions: The results suggest that adding LEAP to a weight management program enhanced participants’ ability to achieve and maintain weight loss. LEAP enabled participants to pursue and achieve their important goals successfully.

## 1. Introduction

Overweight and obesity are defined as excessive or abnormal fat accumulation, which may cause health-related problems and reduce a person’s lifespan. Obesity represents an increasing global epidemic [1,2]. It is the fifth leading cause of death worldwide. Each year, at least 2.8 million adults die because of problems associated with being overweight or obese. Overweight and obesity result from complex interactions among various genetic, physiological, psychological, and environmental factors, which have many psychological and physical consequences [3]. It has also been shown that women are more likely than men to suffer from obesity [4,5].

Interventions for being overweight or obese require a combination of approaches to improve the person’s diet and physical activity and address various psychological factors. Customary weight loss programs include dieting and exercise [6]. Successful weight loss programs should, however, also promote healthy lifestyles so that overweight individuals can lose weight safely and maintain weight loss. There is ample evidence that losing weight is difficult for most individuals. Collectively, these studies support the potential of motivational interventions, particularly when combined with exercise and dietary modifications, for promoting weight loss and improving psychological well-being. Jacob et al.’s [7] meta-analysis underscores the broader effectiveness of cognitive–behavioral interventions for weight management and psychological health. Weinreich et al.’s [8] research demonstrates the potential of integrating psychological components into lifestyle interventions, while Butryn et al.’s [9] review emphasizes the value of behavioral treatments in addressing obesity. These findings highlight the importance of incorporating motivational strategies to enhance the effectiveness of exercise-based weight loss programs and improve long-term outcomes for individuals seeking to manage their weight. Moreover, there is a strong likelihood that lost weight will be regained [10]; therefore, the effects of weight loss programs may not persist over the long term [11]. More than 50% of the people who start an exercise program seem to lose their motivation and usually drop out within three to six months [12,13]. Similarly, most dieters abandon their program after weight loss, causing them to regain the lost weight [14,15,16] 

Overweight individuals cite various factors that hinder their weight management, including both psychological (e.g., negative emotions, depression, stress, lack of motivation, and inconsistency in adhering to their exercise routine) and situational (e.g., tempting food commercials and the easy availability of unhealthy food and drinks) barriers [17]. There are other reasons, such as a lack of information about effective methods for losing weight or the calorie content of various foods [18,19,20]. Therefore, a psychological intervention should be a major component of an effective weight management program [21]. It is very important, moreover, for treatment gains to be maintained over the long term. Considering the multi-dimensional nature of being overweight or obese [3] and the high failure rates of obesity treatments (dieting, exercise), multidisciplinary programs would be expected to improve the success of weight loss programs [22,23,24,25]. 

To better help overweight people, dieting, exercise, and psychoeducational interventions must be combined. An important addition to such an intervention would be a motivational intervention focusing on people’s goal choices and how they pursue their goals. Different people have diverse ways of choosing and pursuing their goals. One factor that affects people’s success or failure at achieving their goals is their motivational structure [26,27,28,29], which can be adaptive or maladaptive [30,31] and is assessed with the Personal Concerns Inventory (PCI) [32,33,34]. An adaptive motivation structure is characterized by expected happiness from goal attainments, choosing goals that have realistic chances of being achieved, and commitment to the pursuits. People’s motivational structure predicts their ability to alleviate problematic behaviors [35]. Therefore, overweight people who can lose weight and maintain the loss in the long term would be expected to have a more adaptive motivational structure.

Cox and Klinger developed Systematic Motivational Counseling (SMC) to assess and change maladaptive motivational patterns into adaptive ones [36,37]. SMC aims to help people lead happier and more fulfilling lives by identifying better ways to resolve their current concerns. SMC has been used for individuals with alcohol and other substance abuse disorders, and evaluations have been consistently favorable [36,37,38]. 

Similarly, the Life Enhancement and Advancement Program (LEAP) was designed as a motivational restructuring technique for helping individuals to find emotional satisfaction without using chemical substances [35,39]. LEAP includes four major components: First, participants are shown how their lack of satisfaction in other areas of their lives can affect their motivation to engage in unhealthy behaviors. Second, they are asked to discuss the goals they named on the PCI, which they completed at the beginning of the program. Third, they complete a goal matrix to assess how each goal facilitates or hinders the achievement of other goals. In turn, they select valued and realistic goals that will not interfere with achieving other goals. Fourth, they specify actions to be taken that will help them reach their chosen goals, and then they develop a plan for achieving each action. In the case of people who use alcohol excessively, LEAP is effective in changing maladaptive behavioral patterns, thereby enabling people to moderate their drinking [35].

Overweight and obesity are multifaceted conditions that place people at risk of developing multiple health problems [40]. There is ample evidence that being overweight is not simply a problem of self-control or a lack of willpower; rather, it is linked to various comorbid conditions [21]. An effective weight loss program requires combining exercise and dietary intervention with a psychological intervention [22,25,41]. Thus, the major goal of the current study was to assess the effectiveness of adding LEAP to a weight management program for overweight and obese females. This goal was achieved by evaluating the different components of the program separately and in combination. Accordingly, there were four groups whose intervention consisted of (a) dieting, exercise, and LEAP; (b) dieting, exercise, and sham training; (c) dieting and exercise; or (d) exercise only. We hypothesized that the group that received the combined dieting, exercise, and LEAP training would show a greater reduction in BMI and WC and greater improvement in adaptive motivation than any of the other three groups. 

## 2. Methods 

### 2.1. Design

We adopted a 4 (groups: (a) exercise, dieting, and LEAP; (b) exercise, dieting, and sham training; (c) exercise and dieting; (d) exercise only) × 3 (time: pre vs. post vs. follow-up) design. The Ethics Committee of the Department of Psychology, Ferdowsi University of Mashhad, approved the study protocol and the data collection procedures on 24 June 2019 (IR.UM.REC.1398.043). 

### 2.2. Sampling, Setting, and Procedure

The convenience sampling method was utilized due to limited resources and time; however, future studies should consider random sampling for greater generalizability. Participants were recruited from one of the local sports centers in the city of Mashhad, Iran. This study was completed within one year after the study adverts were distributed, upon which the follow-up assessment was completed. After announcing this study at a local sports center, potential participants interested in this study contacted the first author (R.M.S., a psychologist with an M.A. degree and sports coaching credentials). She then made the initial contact with the volunteers. To each participant, the goals and method of the research were carefully explained, and she was assured of the confidentiality and anonymity of the data and that participants would be provided with the study results. Each participant then provided written information consent before participation. Next, she conducted the initial eligibility screening using self-reported information and targeted questions. Each participant was randomly assigned to one of the four groups: Group 1: exercise, dieting, and LEAP; Group 2: exercise, dieting, and sham training; Group 3: exercise and dieting; Group 4: (d) exercise only. All four groups attended six of the 45-to-55 min sessions of an exercise program with three sessions per week, each supervised by the same coach and with a target heart rate of 40% to 60% of maximum heart rate. The three groups trained in dieting were referred to a physician nutrition specialist (PNS) in Mashhad, Iran to participate in a dieting program supervised by the second author (M.N.). The group that received the LEAP intervention [39] (see Table 1) had six 2 h sessions conducted by the first author. She was supervised by the fourth author (J.S.F., Full Professor of Psychology with extensive clinical experience). The group that received sham training attended six weekly 2 h classes in learning English. 

The participants’ height was recorded at baseline, and their weight, waist circumference, and BMI were measured at baseline, post-intervention, and three-month follow-up. All participants completed the PCI at all three assessments. Participants’ demographic characteristics, such as age, education level, history of dieting failures, and weight changes during the past three months, were recorded at baseline.

The group that received sham training was instructed in basic reading skills according to the International English Language Testing System (IELTS). The course involved identifying the main components of texts and paragraphs, such as introduction, conclusions, and keywords. This group was also informed that the sessions were designed to assess their reading skills and that no oral or written exams would be required. 

Following completion of this study, participants in the three groups that had not had the LEAP intervention could participate in LEAP sessions if they wished.

### 2.3. Participants

Only females were recruited because the incidence of obesity is higher among females than males [42]. Inclusion criteria were as follows: female gender, 18-to-45 years old (because BMI cannot be assessed in people younger than 18), BMI ≥ 25, willingness to lose 5-to-7% in body weight, ability to understand and communicate in Persian, having given written informed consent. Exclusion criteria were as follows: body weight greater than 150 kg, pregnant or planned pregnancy within three months, currently seeing a dietitian or participating in a weight loss program, weight loss or gain of more than 3% of body weight within the previous month, physical or psychological condition within the past year that would interfere with the ability to undergo the intervention (e.g., backache or knee pain, eating disorder, depression). Ultimately, no participants were excluded from this study.

### 2.4. Outcome Measures

*Personal Concerns Inventory.* The PCI measures participants’ motivational structure [32,34]. Respondents are encouraged to consider their major concerns, wishes, and goals. They then rank each goal (from 0 to 10) on various scales that depict the important dimensions of the goal pursuit (e.g., appetitive or aversive valence, commitment, control, realistic plans) and their expectation from goal achievement (e.g., optimism, emotional satisfaction). In turn, PCI indices are derived from the ratings that indicate the degree to which the motivational structure is adaptive versus maladaptive. An adaptive motivational structure is characterized by having appetitive goals to which the person feels committed to pursue and from which they expect to derive emotional satisfaction and have realistic chances of success. The reliability and validity of the PCI have been confirmed [34,43]. Fadardi et al. developed a summary score that measures adaptive motivation and showed that the summary scores are highly correlated (*r* = 0.98) with PCI adaptive motivation derived from factor analysis or principal component analysis [44]. 

*Body weight.* While dressed in light clothing, each participant’s body weight was measured to a precision of 100 g using a Seca digital scale.

*Height.* While standing barefoot against a wall, each participant’s height was measured using a portable stadiometer (OTM; Tehran, Iran) to a precision of 0.10 cm. 

*Body mass index.* Body mass index (BMI) is a commonly used index of height and weight. It is calculated by dividing weight (in kg) by height (m^2^) [45].

### 2.5. Bias Control

**Figure 1:**
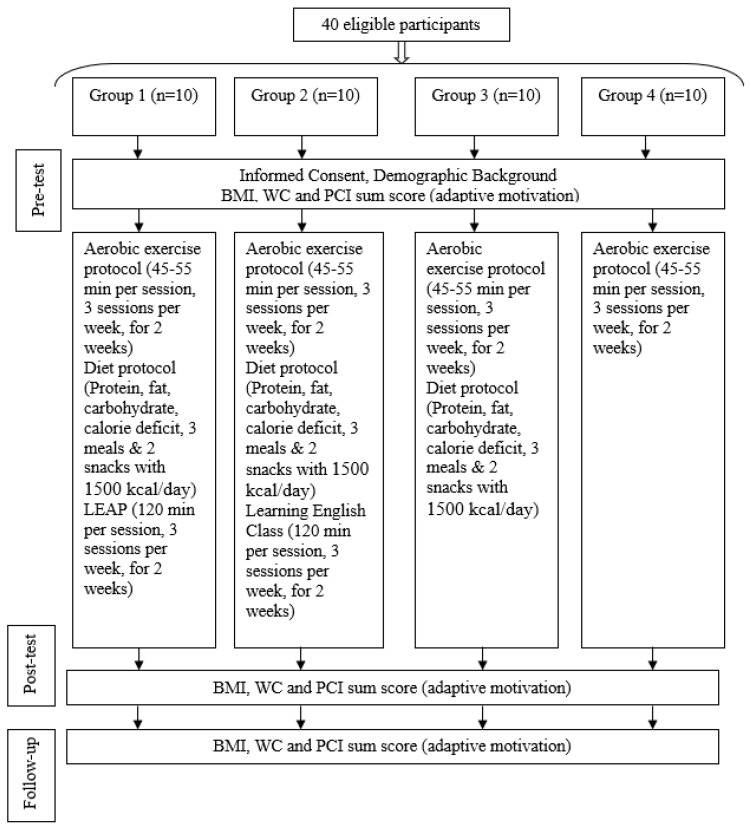
Flow diagram of this study’s procedure. Note. Group 1: exercise, dieting, LEAP; Group 2: exercise, dieting, English class; Group 3: exercise, dieting; Group 4: exercise. BMI: body mass index; WC: waist circumference; PCI: Personal Concerns Inventory; posttest assessment was conducted after a two-week intervention; follow-up assessment was conducted three months post-intervention.

This study followed a single-anonymized design because the first author responsible for data collection was aware of participants’ group allocation; therefore, the outcomes should be interpreted cautiously. We took a few steps to control other potential sources of bias. The reason for the absence of a diet-only group is that all participants were recruited from a sports center whose aim was to lose weight through exercise. It could cause resistance to participation if they were invited to follow a diet-only routine, which could bias the study outcomes. Dietary compliance was monitored using a multifaceted approach based on PNS protocol, including self-reported dietary logs, validated food frequency questionnaires, and regular consultations. These measures allowed for ongoing assessment of adherence to the prescribed dietary guidelines, providing feedback and support to participants throughout the intervention period. Although non-compliance was an exclusion criterion, all participants maintained satisfactory adherence levels. The goal of having sham training was to control for the effects of attention and social interaction. A flow diagram of this study is shown in Figure 1. While initial weight loss may be evident within two weeks, a three-month follow-up is essential to assess the long-term sustainability of weight loss results and their impact on overall lifestyle behaviors. A three-month follow-up aligns with established research practices, such as the Diabetes Prevention Program (DPP), demonstrating the importance of sustained weight loss at three months for long-term diabetes prevention [46]. Additionally, research in obesity highlights that weight regain is common within the first few months after initial weight loss, making the 3-month mark a critical point for evaluating the efficacy of interventions and identifying factors that predict long-term success [9].

### 2.6. Sample Size

Although convenience sampling was used due to practical constraints, estimating the required sample size through a power analysis is recommended to ensure sufficient statistical power. Using G*Power 3.0.3 software and considering effect size estimates (f^2^ = 0.28) from similar studies [47,48,49], with α = 0.05, power = 0.80, number of groups = 4, number of predictors = 6 (four groups and two covariates), and number of response variables = 2, a minimum sample size of 37 was calculated. To account for potential attrition, we aimed to recruit 40 obese or overweight females (BMI ≥ 25 kg/m^2^) from a pool of 60 volunteers at a local sports center. 

### 2.7. Analysis

Analyses included descriptive statistics (e.g., means and standard deviations) and inferential statistics. MANCOVA and Bonferroni post-hoc and paired *t*-tests were used to compare the four groups on the various dependent variables. All data were checked for normality using Q-Q plots [50]. A *p*-value of ≤ 0.05 was set for all analyses.

## 3. Results

Participants’ demographic characteristics, including their education, age, body weight, BMI, WC, history of dieting failures, and weight changes during the previous six months are shown separately for the four groups in Table 2. 

The means and standard deviations of participants’ BMI, WC, and PCI adaptive motivation summary scores at baseline, post-intervention, and three-month follow-up are shown in Table 3. Three separate MANCOVAs were conducted to test the hypothesis that the group receiving exercise, dieting, and LEAP would show a greater reduction in BMI and WC and a greater improvement in PCI adaptive motivation than any of the other three groups. In each MANCOVA, BMI, WC, or PCI adaptive motivation for the post-intervention and the three-month follow-up was entered as the dependent variable, with as the predictor variable. The covariates were participants’ age and education and their baseline BMI, WC, and PCI adaptive motivation scores. 

As Table 4 shows, the results of the first MANCOVA showed a significant overall effect for Group 1 on participants’ BMI with a large effect size, *d* = 0.95 (eta squared effect sizes were converted to Cohen’s *d*, whose values are 0.20, 0.50, and 0.80, for small, medium, and large effects, respectively). Group 1 was not significant at post-intervention, but it was significant at the three-month follow-up. Bonferroni tests indicated that Group 1 (exercise, dieting, and LEAP) was significantly lower on BMI than Group 2 (*p* = 0.005), Group 3 (*p* < 0.001), and Group 4 (*p* < 0.001) at the follow-up; no other group differences were found. 

The results of the second MANCOVA showed a significant overall effect for Group 1 on participants’ WC with a large effect size, *d* = 2.43. The Group 1 effect was significant at both post-intervention and the three-month follow-up. A Bonferroni test indicated that Group 1 had significantly lower WC measurements than Group 2 (*p* < 0.001), Group 3 (*p* < 0.001), and Group 4 (*p* < 0.001) at the follow-up; no other group differences were found.

Finally, the third MANCOVA showed a significant overall effect for Group 1 on participants’ PCI adaptive motivation scores with a large effect size, *d* = 1.67. There was also a significant effect for Group 1 at both post-intervention and the three-month follow-up. A Bonferroni test indicated that the adaptive motivation of Group 1 had significantly improved at the follow-up compared to Group 2 (*p* < 0.001), Group 3 (*p* = 0.01), and Group 4 (*p* < 0.001); nonetheless, the difference between other groups was not significant.

Next, paired *t*-tests were calculated to identify the source of Group 1 X Time interactions on the PCI indices. The results showed improvements in the PCI indices in Group 1 (exercise, dieting, and LEAP), including moral rightness of goal pursuits, plans for goal pursuits, commitment to goal pursuits, and optimism about goal achievements (see Table 5). Participants’ PCI indices in the other three groups did not change significantly from baseline to the other two assessment points. These results, therefore, supported this study’s hypotheses.

At follow-up, participants in Group 1 (exercise, dieting, and LEAP) reported continuing to pursue their new healthy lifestyle, which included exercising and dieting. This further indicates the success of the intervention that Group 1 received.

## 4. Discussion 

This study aimed to assess the effectiveness of LEAP as an add-on intervention to exercise and dieting in a weight management program for overweight and obese females. It is important to acknowledge that this study utilized a convenience sampling method, which may limit the generalizability of the findings to the broader population. While convenience sampling offers practical advantages in feasibility and recruitment, it can introduce potential bias due to the non-random selection of participants. Therefore, caution should be exercised when extrapolating the results beyond the specific characteristics and context of the sampled population. The results supported the hypothesis that *Group 1* (exercise, dieting, and LEAP) would be more successful in helping participants to lose weight than would *Group 2* (exercise, dieting, and sham training to control for the attention that Group 1 received), *Group 3* (exercise and dieting), or *Group 4* (exercise only). Unlike any of the other three groups, the intervention in Group 1 enabled participants to reduce their BMI and WC to maintain the reductions for at least three months. 

Previous studies have shown that interventions for weight management that include both exercise and dieting are more effective than only one of these components [51,52], and the inclusion of a psychological intervention can improve the results even further [3,53]. The results of the present study are also consistent with previous studies that have demonstrated the effectiveness of both *Systematic Motivational Counseling* and the related intervention *Life Enhancement and Advancement Program (LEAP)* for improving treatment outcomes [35,37,38].

In the current study, Group 1 (exercise, dieting, and LEAP) showed other positive outcomes that can be attributed to the effects of LEAP on improving participants’ adaptive motivation. For example, Group 1 showed significant improvements from baseline to post-treatment and from baseline to the follow-up in participants’ (a) willingness to seek information that would facilitate their goal attainments, (b) commitment to achieving their goals, (c) optimism about reaching their goals, and (d) expected chances of success in reaching their goals. Participants in Group 1 were instructed to take an appetitive approach to pursuing their goals, thereby focusing on positive things they would like to achieve instead of negative things they wanted to avoid or get rid of. This attitude, in turn, seemed to strengthen their commitment to goal pursuits [54]. The participants also appeared more likely to seek information to help them achieve their goals than participants in the other three groups. They seemed to become more adept at planning and scheduling their activities that would facilitate their goal attainments and more adept at asking for help when they needed it. As a result, they felt more in control, more committed to their goal pursuits, and more optimistic about reaching their goals. Because participants in Group 1 were instructed to adopt an adaptive motivational approach in their weight loss program, they seemed to derive greater satisfaction from their goal achievements than participants in the other three groups. Finally, participants in Group 1 seemed to believe in the importance of their own role in achieving their weight loss goals. These differences among the participants in the four intervention groups were confirmed by improvements only in Group 1’s motivational structure, which became more adaptive during this study. 

An adaptive motivational structure is characterized by having appetitive goals that the person wants to achieve, knowing the steps to take to reach one’s goals, commitment to and a sense of control over goal pursuits, expectations of success in reaching one’s goals, and expected emotional satisfaction upon reaching one’s goals [32]. From the current study, we conclude that participants in Group 1 (exercise, dieting, and LEAP) achieved more adaptive motivation from having participated in LEAP. It is designed to replace participants’ maladaptive motivational structure with a more adaptive one. The LEAP component of the intervention in Group 1 focused largely on participants’ motivational structure, rather than solely on exercise or dieting. Using LEAP, we initially assessed the characteristics of the goals that the participants named (e.g., the degree to which they were appetitive or aversive) and then tried to shift them from aversive to appetitive goals. We did so by using strategies such as goals prioritization, enhancing commitment to realistic and achievable goals, finding solutions to conflicts among different goals, and identifying realistic steps to goal attainment.

The overarching goal of LEAP was to promote participants’ happiness and satisfaction by maximizing their success in reaching attractive and achievable goals. Research has shown that life satisfaction is achieved by having positive outcomes in various life domains [55]. Life satisfaction derived from achieving small goals is key to motivating people to modify their life circumstances by working to achieve their personal goals. By the end of the LEAP sessions, the participants seemed to have more realistic beliefs about weight loss, and their motivation for weight loss appeared to be more internalized, whereby they sought to lose weight to promote their long-term health.

The current study utilized novel techniques to evaluate the cumulative effects of weight loss components (LEAP, dieting, and exercise) for overweight and obese females. The minimum and maximum weight loss was achieved, respectively, in Group 4 (exercise only) and in Group 1 (exercise, dieting, and LEAP), thereby confirming the greater effectiveness of the intervention that Group 1 received. We used the sham training in Group 2 (exercise, dieting, English class) to control for the additional attention that Group 1 (exercise, dieting, LEAP) received. As noted, the improvements in Group 1 can be attributed to the specific effects of LEAP rather than the supportive–expressive effects of being in the group. Moreover, the LEAP might fortify the effects of dieting and exercise by addressing motivational components, especially those significantly improved post-training (i.e., moral rightness, plans for goal pursuits, commitment to goal pursuits, and optimism about goal achievements).

While BMI is a widely used and convenient measure, it does not differentiate between fat and lean mass, potentially leading to misclassification in individuals with high muscle mass [45]. The lack of significant group differences between the exercise-only group and the exercise plus diet groups in this study, as indicated by BMI and WC, might be attributed to this limitation of BMI. It is plausible that participants in the exercise-only group experienced increased muscle mass that offset any potential fat loss, resulting in minimal change in BMI despite improvements in body composition.

Incorporating measures such as waist-to-hip ratio (WHR) and body fat percentage would provide a more comprehensive assessment of body composition and adiposity distribution, offering valuable insights into the health risks associated with obesity [42]. These measures could potentially reveal differences between groups not captured by BMI alone. For example, the exercise-only group might show decreased body fat percentage or a more favorable WHR than the exercise plus diet groups, even if their BMI remained relatively stable.

This study primarily focused on BMI and WC due to their established associations with cardiometabolic risk factors and their widespread use in clinical practice [3]. However, we recognize the value of including additional measures like WHR and body fat percentage in future research to enhance the understanding of the impact of lifestyle interventions on body composition and overall health. Notwithstanding the strengths of the present study, there were also some limitations. First, we selected participants from clients enrolling with a sports center in Mashhad, and the sampling method might limit the generalizability of the results. Second, the follow-up assessment (three months) was relatively short. Despite the favorable outcomes among participants in Group 1 (exercise, dieting, LEAP), caution should be exercised in drawing definitive conclusions from the results. Accordingly, larger studies with a more diverse sample (e.g., male and female participants) and a longer follow-up period (e.g., 12 months) are warranted. Finally, future research would benefit from comparing LEAP with other weight management interventions and psychological approaches to augment the effects of exercise and diet in achieving weight loss.

## 5. Conclusions

Although dieting and exercise can have short-term effects on weight loss, it is essential to focus on broader motivational issues to help participants achieve long-term effects. The present study confirmed that motivational training in addition to dieting and exercise improved female participants’ ability to lose weight by enhancing their adaptive motivation. The results of this study point to the need for additional research on the use of dieting, exercise, and LEAP in weight management for overweight and obese females.

## Figures and Tables

**Table 1:** Steps in the Life Enhancement and Advancement Program (LEAP) used in this study.

Session	Goal	Topics	Task
1	Introduction to LEAP	Established rapport. Explained the rationale and method of LEAP; the importance of goals and their successful pursuit for emotional satisfaction; how LEAP can improve people’s quality of life and happiness. Discussed the nature of people’s goals and goal-seeking activities and adaptive and maladaptive motivational structures. Introduced the Personal Concerns Inventory (PCI) and how it should be completed.	Administered the PCI
2	How to score and interpret the PCI	Helped participants complete and score the PCI and interpret the results. Discussed adaptive vs. maladaptive motivation, different motivational profiles, and how to achieve adaptive motivation, e.g., by taking a more active role in goal pursuits, examining sources of control, and planning how to pursue specific goals, such as weight control. Identified new incentives and goals for participants to pursue and helped them to disengage from inappropriate goals.	Worked on enhancing participants motivation. Completed the Satisfaction with Life Scale (SWLS) and Dutch Eating Behavior Questionnaire (DEBQ).
3	Helped to improve participants’ knowledge about and sense of control over their eating behavior	Discussed general and personal causes of overeating and consuming too many high-calorie foods. Compiled a list of false beliefs vs. facts about eating high-calorie foods. Identified situations that motivate the consumption of high-calorie foods (why, when, where, how, with whom).	Provided a list of personal advantages and disadvantages of weight control.
4	Promoted goal commitment by clarifying the link between lifestyle, weight control, and resulting gratification	Explained different eating styles (emotional, external, inhibited) and how they are related to food consumption. Discussed realistic weight control targets and the relationship between weight control and having a happier lifestyle. Explained three general methods for achieving a happier lifestyle. Identified individual sources of happiness and stress and ways to reduce daily life stresses. Explored alternative activities to the consumption of high-calorie foods.	Identified the sources of daily feelings related to overeating; focused on activities that can reduce food intake.
5	Reviewed participants’ goals and ways to achieve appropriate goals	Reviewed participants’ personal goals that might increase their happiness. Focused on both the quantity (using a goal matrix) and quality of participants’ goals and the nature of people’s satisfaction upon achieving their goals. Discussed how other goals impact the goal of losing weight; the importance of goal planning based on each participant’s PCI results.	Completed a list of things that are important to me; completed a goal matrix and a sample goal ladder.
6	Learned how to design a goal ladder	Developed a plan for achieving the goal of losing weight. Introduced SMART (specific, measurable, attainable, relevant, time-bound) goals and the prioritization of goals. Designed a goal ladder and prioritized steps for losing weight (eating less, changing bad eating habits, reducing the size of meals, increasing the number of meals, increasing physical activity). Discussed specific steps for achieving these goals. Identified potential obstacles and ways to overcome them. Designed a target ladder for reading a specific weight loss goal. Obtained feedback from other participants.	Reviewed the sessions and drew conclusions. Identified dates for the follow-up assessments.

Note. At the beginning of each session, participants’ homework from the previous session was checked and discussed.

**Table 2:** Participants’ demographic characteristics.

		Group							
		Group 1 (*n* = 10)		Group 2 (*n* = 10)		Group 3 (*n* = 10)		Group 4 (*n* = 10)	
		%		%		%		%	
Education									
	<High school	18.2%		9.1%		45.5%		27.3%	
	High school	27.3%		27.3%		0.00%		0.00%	
	Associate’s degree	27.3%		0.00%		0.00%		36.4%	
	Bachelor’s degree	27.3%		54.5%		45.5%		18.2%	
	Master’s degree	0.00%		0.00%		0.00%		9.1%	
		M	sd	M	sd	M	sd	M	sd
Age		30.00	7.94	27.80	9.72	32.30	9.09	29.90	7.62
Weight (kg.)		81.27	11.59	72.20	6.86	77.10	12.11	76.7	9.49
Height (cm.)		1.64	7.00	1.57	7.10	1.61	6.15	1.59	5.40
BMI		29.68	3.31	29.30	3.06	29.28	3.07	30.28	3.30
WC (cm.)		101.95	4.07	102.56	4.35	100.89	5.15	101.62	4.20
No. of dieting failures		2.90	2.94	2.50	2.32	1.80	2.04	3.10	2.33
Weight changes during last 6 months (kg.)		−0.27	2.28	−0.10	3.21	−0.20	3.58	1.00	5.33

Note. Group 1: exercise, dieting, LEAP; Group 2: exercise, dieting, English class; Group 3: exercise, dieting; Group 4: exercise.

**Table 3:** Means and standard deviations of participants’ BMI, WC, PCI adaptive motivation scores.

		BMI		WC		PCI	
		M	sd	M	sd	M	sd
Group 1	Pretest	29.68	3.31	101.95	4.07	54.71	2.37
	Posttest	28.52	3.53	97.13	5.03	62.30	1.83
	Follow-up	26.50	2.34	88.95	3.97	62.63	1.88
Group 2	Pretest	29.30	3.06	102.56	4.35	55.72	2.07
	Posttest	28.001	2.58	101.88	4.77	55.26	2.33
	Follow-up	27.99	2.52	101.19	4.83	54.76	2.401
Group 3	Pretest	28.93	3.31	100.89	5.15	54.83	2.63
	Posttest	28.00	3.05	100.00	5.35	55.007	2.11
	Follow-up	28.68	3.09	99.16	5.19	54.66	2.17
Group 4	Pretest	29.01	3.62	101.62	4.20	54.89	2.82
	Posttest	29.10	3.37	100.56	3.81	54.34	2.11
	Follow-up	29.08	3.52	100.90	6.84	54.26	2.17

Note. Group 1: exercise, dieting, LEAP; Group 2: exercise, dieting, English class; Group 3: exercise, dieting; Group 4: exercise.

**Table 4:** MANCOVA results for the effects of group on BMI, WC, and PCI adaptive motivation.

Outcome Variable	Overall Effect F(6, 56) [*d*]	Post-Intervention F(3, 36) [*d*]	Follow-Up F(3, 36) [*d*]	Post-Hoc Comparisons (Bonferroni)
BMI	7.05 ** [0.95]	0.72 [0.31]	17.79 ** [1.52]	Group 1 < Group 2, 3, 4
Waist circumference (WC)	45.40 ** [2.43]	14.72 ** [1.38]	31.20 ** [2.01]	Group 1 < Group 2, 3, 4
PCI adaptive motivation	21.57 * [1.67]	3.69 * [0.69]	17.79 ** [1.52]	Group 1 > Group 2, 3, 4

Note. Group 1: exercise, dieting, LEAP; Group 2: exercise, dieting, English class; Group 3: exercise, dieting; Group 4: exercise; *d* = Cohen’s *d*, calculated from eta squared effect sizes. *p*-values = ** *p* < 0.01; * *p* < 0.05.

**Table 5:** Results of paired *t*-tests of PCI scores at baseline, post-intervention, and follow-up in Group 1.

PCI Index	Time	*p*	*t*_(9)_
Moral rightness	T_1_–T_2_	0.01	3.02
	T_1_–T_3_	0.01	3.11
Plans for goal pursuits	T_1_–T_2_	0.04	2.22
	T_1_–T_3_	0.04	2.20
Commitment to goal pursuits	T_1_–T_2_	0.001	4.45
	T_1_–T_3_	0.001	4.52
Optimism about goal achievements	T_1_–T_2_	0.04	2.31
	T_1_–T_3_	0.02	2.54

Notes. There was no significant change in the other PCI indices across the assessment points, including T_2_–T_3_. Group 1: exercise, dieting, and LEAP.

## Data Availability

Data are contained within the article. Additional data are unavailable due to privacy and ethical restrictions.
